# Coaxial electrospinning of poly(ɛ-caprolactone)/gelatin core-shell biodegradable implants for localized delivery of metronidazole and dexamethasone for periodontal applications

**DOI:** 10.3389/fbioe.2026.1770271

**Published:** 2026-05-22

**Authors:** Sarah Chang, Caroline Anselmi, Igor Mendes Soares, Lais Medeiros Cardoso, Alexandre Henrique dos Reis-Prado, Ana Beatriz Gomes de Carvalho, J. Christopher Fenno, Renan Dal-Fabbro, Josimeri Hebling, Marco C. Bottino

**Affiliations:** 1 Department of Cariology, Restorative Sciences, and Endodontics, School of Dentistry, University of Michigan, Ann Arbor, MI, United States; 2 Department of Morphology and Pediatric Dentistry, School of Dentistry, São Paulo State University (UNESP), Araraquara, Brazil; 3 Department of Dental Materials and Prosthodontics, School of Dentistry, São Paulo State University (UNESP), Araraquara, Brazil; 4 Department of Restorative Dentistry, School of Dentistry, Federal University of Minas Gerais (UFMG), Belo Horizonte, Minas Gerais, Brazil; 5 Department of Dental Materials and Prosthodontics, School of Dentistry, São Paulo State University (UNESP), São José dos Campos, Brazil; 6 Department of Biologic and Materials Sciences and Prosthodontics, School of Dentistry, University of Michigan, Ann Arbor, MI, United States; 7 Department of Biomedical Engineering, College of Engineering, University of Michigan, Ann Arbor, MI, United States

**Keywords:** core-shell, dexamethasone, gelatin, guided tissue regeneration, metronidazole, periodontal tissue regeneration, polycaprolactone, tissue engineering

## Abstract

**Introduction:**

Core-shell electrospun scaffolds may help coordinate antibacterial and immunomodulatory effects during periodontal healing. Their unique architecture may enable a more spatiotemporal control over the delivery of therapeutic agents. This study fabricated coaxial poly(ε-caprolactone)/gelatin (PCL/Gel) fibers loaded with dexamethasone (DEX) and metronidazole (MET) and evaluated their physicochemical and biological performance.

**Methods:**

PCL/Gel core-shell fibers were electrospun with DEX (5% w/w in the PCL core) and MET (30% w/w in the gelatin shell), producing four groups: PCL/Gel (control), MET, DEX, and DEX/MET. Core-shell structure and morphology were assessed by SEM, TEM, and fluorescence imaging; mass loss in PBS and tensile properties were measured. Antibiofilm activity against *Porphyromonas gingivalis* (Pg), alveolar bone-derived mesenchymal stem cells (aBMSCs) viability/spreading and mineralization, and IL-1α/TNF-α production by LPS-stimulated RAW 264.7 macrophages exposed to scaffold extracts were evaluated. Data were analyzed based on normality and variance; group comparisons used one- or two-way ANOVA with appropriate *post hoc* tests (α = 0.05).

**Results:**

All groups formed uniform core–shell fibers with comparable degradation and mechanical behavior. MET-containing scaffolds reduced Pg biofilm recovery (p ≤ 0.032). After LPS stimulation, IL-1α levels returned to baseline only in DEX-containing groups (DEX and DEX/MET), whereas IL-1α remained elevated in groups without DEX (p ≤ 0.0119); TNF-α was also significantly reduced in DEX-containing groups versus control and MET (p < 0.0001). All scaffolds supported increasing aBMSCs viability and spreading; mineralization increased from day 14–21 in all groups (p < 0.0001), with the highest deposition in the DEX-only group at day 21 (p ≤ 0.0032).

**Conclusion:**

Coaxial PCL/Gel core-shell fibers provided antibiofilm and anti-inflammatory functionality while maintaining cytocompatibility and osteogenic compatibility, supporting their potential as a multifunctional scaffold for periodontal regenerative applications.

## 1 Introduction

Periodontitis is a prevalent multifactorial disease characterized by microbial dysbiosis and a dysregulated inflammatory host response (Van Dyke et al., 2025). Endotoxins and metabolites produced by periodontal pathogens can sustain oxidative stress and disrupt cytokine homeostasis; however, disease progression is ultimately dictated by the host immune response, culminating in irreversible destruction of periodontal tissues and, in severe cases, tooth loss due to alveolar bone resorption (Suarez et al., 2020; Van Dyke et al., 2025). Current clinical management primarily focuses on controlling pathogenic biofilms through mechanical debridement of plaque and calculus (Jervoe-Storm et al., 2022). Nonetheless, achieving predictable periodontal regeneration after advanced disease remains challenging because periodontal tissues have limited intrinsic regenerative capacity and the local microenvironment often persists in a chronic inflammatory state (Wen et al., 2024). Therefore, smart local-delivery systems capable of providing antibiotics and immunomodulators have been proposed to help restore a pro-regenerative milieu in periodontal defects (Daghrery and Bottino, 2022; Li et al., 2024; Aytac et al., 2022; Bottino et al., 2017b; Bottino and Thomas, 2015; Bottino et al., 2011).

A therapeutic approach that combines adjunctive medications is particularly attractive because it can achieve early infection control, followed by resolution of inflammation and tissue repair (Bottino et al., 2017b; Bottino et al., 2014; Bottino et al., 2017a; Li at al., 2024). In this context, metronidazole (MET) and dexamethasone (DEX) are compelling candidates. MET, a nitroimidazole antibiotic, suppresses anaerobic pathogens commonly implicated in periodontitis (Sgolastra et al., 2014; Miguel et al., 2025), whereas DEX, a synthetic glucocorticoid, modulates inflammatory signaling and can promote osteoblastic differentiation, supporting mineralized tissue formation (Bordini et al., 2021; Noory et al., 2025). Nanofibrous scaffolds have emerged as effective platforms for localized and controlled drug delivery because their extracellular-matrix-like architecture promotes favorable interactions with periodontal tissues and enables prolonged local retention (Bottino et al., 2017b). However, conventional electrospun fibrous systems often exhibit an initial burst and largely simultaneous release of encapsulated agents (Cho et al., 2025), potentially exposing surrounding cells to cytotoxic concentrations and limiting sustained therapeutic efficacy. In previously reported non-coaxial dual-drug systems, the lack of spatial segregation within the fiber matrix limits independent modulation of the release kinetics of each therapeutic agent.

Core-shell fibers produced by coaxial electrospinning enable spatial and temporal control over drug release, helping address these limitations (Abdullah et al., 2019; Cho et al., 2025). By compartmentalizing drugs within distinct fiber layers, this architecture enables rapid release from the shell while sustaining core delivery, improving control over sequential and sustained therapeutic presentation compared with conventional electrospun and non-coaxial dual-drug systems (Cho et al., 2025). Such systems can synchronize drug availability with the biological stages of healing, optimizing infection control and tissue regeneration (Abdullah et al., 2019). Polymer selection is central to this design, as degradation rate, hydrophilicity, and mechanical behavior directly influence diffusion, release kinetics, and cell-material interactions. Pairing a relatively fast-degrading, hydrophilic shell with a more stable core can thus provide tunable release profiles while supporting cell adhesion, proliferation, and function (Abdullah et al., 2019).

Poly(ɛ-caprolactone) (PCL) is a cost-effective, biodegradable synthetic polymer recognized for its biocompatibility, mechanical strength, and slow degradation rate, making it an ideal core material. Despite its favorable mechanical performance, PCL’s hydrophobicity limits cell adhesion, motivating its combination with natural polymers to enhance bioactivity (Siddiqui et al., 2018; Ramírez-Ruiz et al., 2025). Gelatin, a naturally derived polymer obtained from collagen, offers high biocompatibility, biodegradability, and cell-recognition sites that support adhesion and proliferation (Echave et al., 2019; Wu et al., 2024). Its hydrophilic and fast-degrading nature makes it suitable as a shell component for early-stage drug release, while crosslinking can further improve mechanical integrity and stability (Echave et al., 2019; Wu et al., 2024).

Despite increasing interest in localized drug-delivery strategies for periodontal therapy, the spatiotemporal co-delivery of MET and DEX from coaxially electrospun fibers remains largely unexplored. A coaxial polycaprolactone/gelatin (PCL/Gel) core-shell design may enable sequential delivery, with rapid MET release from the gelatin shell to provide early antibacterial activity and sustained DEX release from the PCL core to support prolonged immunomodulatory and osteogenic effects. This controlled dual-release profile is expected to coordinate antimicrobial, immunomodulatory, and reparative events, enhancing periodontal tissue regeneration. Therefore, this study hypothesized that coaxial PCL/Gel scaffold implants co-delivering MET and DEX would exert synergistic antibacterial, anti-inflammatory, and osteogenic effects, creating a microenvironment conducive to periodontal mesenchymal stem cell function without compromising the fibers’ physicochemical properties.

## 2 Materials and methods

### 2.1 Fabrication and characterization of PCL/Gel core-shell scaffold

**FIGURE 1:**
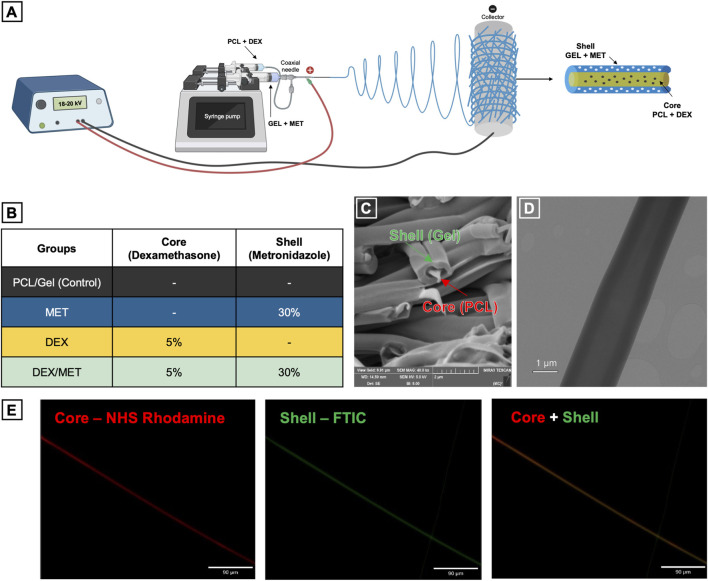
**(A)** Schematic representation of electrospinning of the core-shell scaffolds; **(B)** Core-shell groups and their respective drug incorporation; **(C)** SEM image of the control scaffold cross-section (40,000x); **(D)** TEM image of a single fiber showing the coaxial structure; **(E)** Fluorescence image of a core-shell fiber stained with NHS - Rhodamine (0.01 mg/mL; red; core) and Fluorescein-5-Isothiocyanate (FTIC; 0.01 mg/mL; green; shell).

Core-shell scaffolds were fabricated by electrospinning. For the core, PCL (10% w/v; Mw 50,000 g/mol; Cellink, Gothenburg, Sweden) was dissolved in 4 mL of 1,1,1,3,3,3-hexafluoroisopropyl alcohol (HFIP; Mw 168.04 g/mol; Chem-Impex, Wood Dale, IL, United States) and magnetically stirred at room temperature for 24 h. For the shell, porcine skin gelatin (10% w/v; Mw 50,000–100,000 g/mol; Sigma-Aldrich, St. Louis, MO, United States) was dissolved in 6 mL HFIP and magnetically stirred at room temperature for 24 h. DEX (5% w/w; Mw 392.47; Thermo Fisher Scientific, Waltham, MA, United States) was added to the PCL solution and stirred at room temperature for an additional 24 h; the dose was selected based on prior mineralization studies (Daghrery et al., 2020). MET (30% w/w; Mw 171.15; Sigma-Aldrich, St. Louis, MO, United States) was added to the gelatin solution and stirred at room temperature for an additional 24 h (Silverberg et al., 2025). Vials were wrapped in aluminum foil for light protection. Four groups were prepared: PCL/Gel (control), MET, DEX, and DEX/MET (Figures 1 A,B). After 48 h total homogenization, each formulation was loaded into a 3-mL syringe (PCL) and a 10-mL syringe (Gel) and co-electrospun using a coaxial needle (26G/14G). Syringes were mounted on a syringe pump (KD Scientific, Holliston, MA, United States) at 0.5 mL/h (PCL) and 1.2–1.4 mL/h (Gel), with 20–22 kV applied. Fibers were collected on a non-stick aluminum rotating collector (120 rpm) positioned 18 cm from the needle tip. Scaffolds were vacuum-dried (Isotemp Model 281A, Thermo Fisher Scientific) for 24 h to remove residual solvent, then gelatin shells were stabilized by chemical crosslinking with EDC (5 mM in 80% v/v ethanol; Mw 191.70; Thermo Fisher Scientific) following a published protocol (Campiglio et al., 2019).

### 2.2 Morphological characterization of PCL/Gel core-shell scaffolds

Scaffold morphology was assessed on cross-sections using scanning electron microscopy (SEM), transmission electron microscopy (TEM), and fluorescence imaging. For each group, 1 × 1 cm samples were mounted on stubs, gold sputter-coated, and imaged in a high-vacuum chamber at 12–15 kV (JMS-6610V, JEOL; Tokyo, Japan). Three fields per sample were captured at 5,000× magnification (n = 12), and fiber diameter was quantified in ImageJ (NIH; Bethesda, MD, United States) by measuring 600 fibers total (50 fibers/image). For fluorescence imaging, the PCL core and gelatin shell were labeled by adding NHS-rhodamine (0.001% w/v; Mw 528; Thermo Fisher Scientific) and FITC (fluorescein-5-isothiocyanate, Isomer I; 0.001% w/v; Thermo Fisher Scientific), respectively, to the polymer solutions and stirring for 30 min at room temperature with vials protected from light. The labeled solutions were electrospun, the fibers were collected on glass, and the samples were imaged using a fluorescence microscope (Echo Revolve, BICO; Lake Zurich, IL, United States).

### 2.3 Physico-chemical characterization of PCL/Gel core-shell scaffolds

Scaffold solubility and *in vitro* degradation were assessed in phosphate-buffered saline (PBS; pH 7.4 ± 0.2; Sigma-Aldrich). Individual scaffolds (n = 6) were immersed in 5 mL PBS and incubated at 37 °C. Initial dry mass (M_0_) was recorded using an analytical balance (0.01 mg readability; Sartorius Quintix 124-1S, Sartorius; Göttingen, Germany). At Day 1, 3, and 5; Weeks 1, 2, and 3; and Months 1 and 2, samples were removed, rinsed with distilled water, vacuum-dried for 24 h, and reweighed (M_x_). Mass loss (%) was calculated as: $100-\left(\frac{M_{x}\times 100}{M_{0}}\right)$ .

The chemical composition was analyzed by FTIR spectroscopy (Frontier, PerkinElmer; UATR accessory; Waltham, MA, United States). Eight groups were evaluated: four powders (PCL, gelatin, DEX, MET) and four scaffold formulations (PCL/Gel, DEX, MET, DEX/MET; n = 3). Spectra were collected in absorbance mode (16 scans/sample, 4 cm^−1^ resolution) over the 2000–400 cm^−1^ range. The mechanical properties of the scaffolds, including Young’s modulus (MPa), tensile strength (MPa), and elongation at break (%), were evaluated using a uniaxial tensile testing machine (Expert 5601, ADMET, Inc.; Norwood, MA, United States) at a speed of 1 mm/h (n = 10). Samples were cut into rectangular strips measuring 25 mm × 3 mm. The width of each strip was recorded as the average of three measurements taken at the top, middle, and bottom using a precision digital caliper with 0.01 mm readability (CD-P6″SWW, Mitutoyo Corp.; Aurora, IL, United States). The gauge length for axial strain measurement was set to 15 mm.

### 2.4 Antibacterial activity of PCL/Gel core-shell scaffolds against *Porphyromonas gingivalis* (Pg)

Antibacterial activity of PCL/Gel core-shell scaffolds against *Porphyromonas gingivalis* (Pg; ATCC 33277; ATCC, Manassas, VA, United States) was evaluated using a biofilm model (de Souza et al., 2025). Scaffolds were cut into 1 × 1 cm^2^ specimens (n = 6), mounted on CellCrown™ plastic devices (Scaffdex, Tampere, Finland), crosslinked, and UV-disinfected for 1 h per side. Samples were transferred aseptically to 24-well plates (Corning, New York, NY, United States) containing 1.8 mL BH-hemin-menadione (BHI-HM) broth supplemented with 1% sucrose (Sigma-Aldrich), then inoculated with 200 μL Pg suspension (1 × 10^8^ CFU/mL). Plates were incubated anaerobically at 37 °C for 3 days. Biofilms were quantified by CFU counting and imaged by SEM (JSM-6610V, JEOL) at 5,000×, 10,000×, and 20,000×.

### 2.5 Alveolar bone-derived mesenchymal stem cells (aBMSCs) response to PCL/Gel core-shell scaffolds

Alveolar bone-derived mesenchymal stem cells (aBMSCs) were isolated previously and verified to express MSC markers (CD73, CD90, CD105) (Mason et al., 2014). Cells were expanded in 75 cm^2^ flasks (Corning, New York, NY, United States) in α-MEM supplemented with L-glutamine, ribonucleosides, deoxyribonucleosides, 15% fetal bovine serum (FBS), and 1% penicillin-streptomycin (all Gibco, Invitrogen, Carlsbad, CA, United States) at 37 °C and 5% CO_2_. At confluence, cells were detached with 0.25% trypsin-EDTA (Gibco) and passaged; passages 4–8 were used for all assays.

Scaffolds were cut into 1 × 1 cm^2^ samples, crosslinked, and UV-disinfected for 1 h per side (n = 10). aBMSCs (3 × 10^4^) were seeded onto scaffolds in 24-well plates (Corning) and cultured in α-MEM with 15% FBS and 1% penicillin-streptomycin (Gibco, Invitrogen; Waltham, MA, United States) at 37 °C and 5% CO_2_. Cell viability was measured at days 1, 3, and 7 using 10% alamarBlue® (Invitrogen) in α-MEM without FBS. After 3 h, fluorescence was measured at 560 nm excitation and 590 nm emission (SpectraMax iD3, Molecular Devices LLC, San Jose, CA, United States) and normalized to blank wells, with blanks set to 100% at each time point.

Cell spreading/adhesion was assessed under the same seeding conditions (n = 4). At days 1, 3, and 7, cells were fixed in 4% paraformaldehyde (Sigma-Aldrich) for 15 min, rinsed twice with PBS, stained with ActinGreen 488 (ReadyProbes; Invitrogen; 1:20 in PBS, 30 min, dark), and counterstained with DAPI (Thermo Fisher Scientific; 1:5,000 in PBS). Samples were imaged by fluorescence microscopy (Echo Revolve, BICO) at 10×. Mineralized matrix deposition was quantified using the same seeding protocol (n = 6). Cells were cultured in osteogenic medium (complete α-MEM plus 0.1 μM dexamethasone, 10 mM glycerophosphate, and 50 μg/mL ascorbic acid) for up to 21 days. On days 14 and 21, samples were fixed in 70% ethanol at 4 °C and stained with 40 mM Alizarin Red (pH 4.2; Sigma-Aldrich) for 1 h, then washed with distilled water until clear. Calcium-rich deposits were imaged by optical microscopy (Echo Revolve, BICO). Bound dye was eluted with 10 mM cetylpyridinium chloride (pH 7.0; Sigma-Aldrich) for 20 min on a shaker, and absorbance was measured at 570 nm (SpectraMax iD3). Day-14 control absorbance was used as the baseline (100%). Cell-free scaffolds processed identically were used for background subtraction.

### 2.6 Anti-inflammatory response of RAW 264.7 macrophages to PCL/Gel core-shell scaffolds

Anti-inflammatory effects of DEX- and MET-loaded core-shell scaffolds were evaluated in RAW 264.7 macrophages (ATCC TIB-71) by measuring cytokine production after *E. coli* lipopolysaccharide (LPS; Sigma-Aldrich) stimulation. Scaffolds were cut into 1 × 1 cm^2^ samples, placed in 24-well plates (Corning), and incubated in DMEM (Gibco) at 37 °C for 24 h to generate scaffold extracts (n = 6), which were collected and stored. RAW 264.7 cells (1 × 10^5^/well) were seeded in separate 24-well plates in DMEM with 10% FBS and cultured at 37 °C, 5% CO_2_. After 24 h, cells were stimulated with 0.1 μg/mL *E. coli* LPS in serum-free DMEM for 6 h; non-stimulated cells served as controls. Cells were then exposed to scaffold extracts for 24 h, after which supernatants were collected and IL-1α and TNF-α were quantified using ELISA MAX Deluxe kits (BioLegend; San Diego, CA, United States) per the manufacturer’s instructions. Absorbance was read at 450 nm (SpectraMax iD3, Molecular Devices LLC).

### 2.7 Statistical analysis

Statistical analyses were selected based on data normality (Shapiro-Wilk) and homogeneity of variance (Levene). Cell viability and antibacterial outcomes across scaffold groups (CT, DEX, MET, DEX/MET) were analyzed by one-way ANOVA with Tukey or Games-Howell *post hoc* tests. Two-way ANOVA was used to assess the effects of time and treatment on mineralized matrix deposition and pro-inflammatory cytokine levels, followed by Sidak *post hoc* testing. Mass degradation was evaluated using 95% confidence intervals. Fiber diameter and mechanical properties (Young’s modulus, tensile strength, and elongation at break) were analyzed using Kruskal–Wallis tests with Dunn’s *post hoc* comparisons. Fluorescence imaging was assessed qualitatively. Sample sizes were set to maintain >80% power at α = 0.05.

## 3 Results

### 3.1 Characterization, degradation, and mechanical properties of DEX/MET-loaded PCL/Gel core-shell scaffolds

**FIGURE 2:**
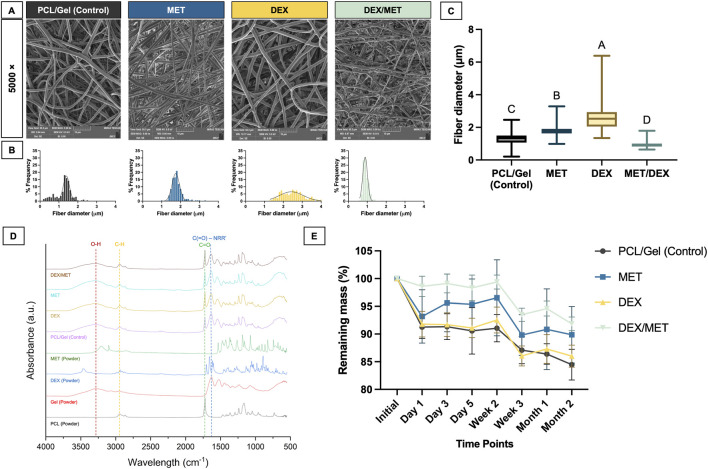
**(A)** SEM images at 5,000× magnification of the surface of crosslinked electrospun scaffolds and histogram of fiber diameter (μm) of crosslinked electrospun scaffolds; **(B)** Fiber diameter frequency histograms (n = 200); **(C)** Boxplots of fiber diameter (median, 25th–75th percentiles). Groups identified by different letters show a statistically significant difference (Kruskal–Wallis, Dunn’s *post hoc* test, α = 5%, n = 200). **(D)** FTIR spectra of PCL, Gel, DEX, and MET powders in comparison with each group of core-shell scaffolds; **(E)** Remaining mass of experimental scaffolds in PBS over 2 months. Values are means and confidence intervals (95% CI, n = 6).

**FIGURE 3:**
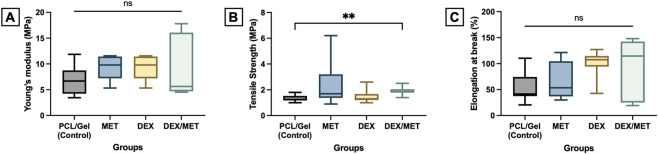
Box plots of the **(A)** Young’s modulus, **(B)** tensile strength, and **(C)** elongation. Values are median, minimum, maximum, 25th, and 75th quartiles. Asterisks indicate significance (Kruskal–Wallis/Dunn’s, α = 5%, n = 10).

SEM and TEM imaging confirmed that all electrospun scaffolds displayed a well-defined core-shell architecture, with PCL forming the core and a surrounding gelatin shell ( Figures 1C-E). According to the TEM images, the core represented 38.63% ± 6.27% of the shell diameter. Across all formulations, fibers appeared continuous and uniformly formed, with no obvious defects or major morphological changes associated with drug incorporation. Quantitative fiber diameter analysis showed that diameters followed a normal distribution in the control, MET, and DEX/MET groups, whereas the DEX group deviated from normality (Figures 2A,B). Fibers incorporated with DEX presented the highest median diameter (2.53 µm), while the combination of both drugs resulted in the lowest median diameter (0.90 µm); intermediate values were observed for the control (1.34 µm) and MET (1.76 µm) groups (Figure 2C). FTIR spectra confirmed the expected chemical composition of the scaffolds, with the presence of gelatin evidenced by the broad O–H/N–H stretching band (∼3,200–3,500 cm^−1^) and characteristic amide-related peaks, alongside the typical absorption bands of PCL (Figure 2D). No additional peaks or significant shifts were observed after drug incorporation, suggesting that the inclusion of DEX and MET did not induce chemical modifications within the system (Figure 2D). *In vitro* degradation in PBS revealed a slow mass-loss profile for all formulations. Over the two-month period, scaffolds retained approximately 80% of their initial dry mass, indicating relatively stable constructs with limited degradation under these conditions (Figure 2E; Supplementary Figure S1B). Mechanical testing showed broadly comparable performance among groups, with similar Young’s modulus, tensile strength, and elongation at break (Figure 3). The only statistically significant difference observed was in tensile strength between the control (CT) and DEX/MET groups (p = 0.0089), with the DEX/MET scaffolds showing a slightly higher mean tensile strength (1.9 MPa) (Figure 3).

### 3.2 Metronidazole-loaded scaffolds suppress *Porphyromonas gingivalis* biofilm formation

**FIGURE 4:**
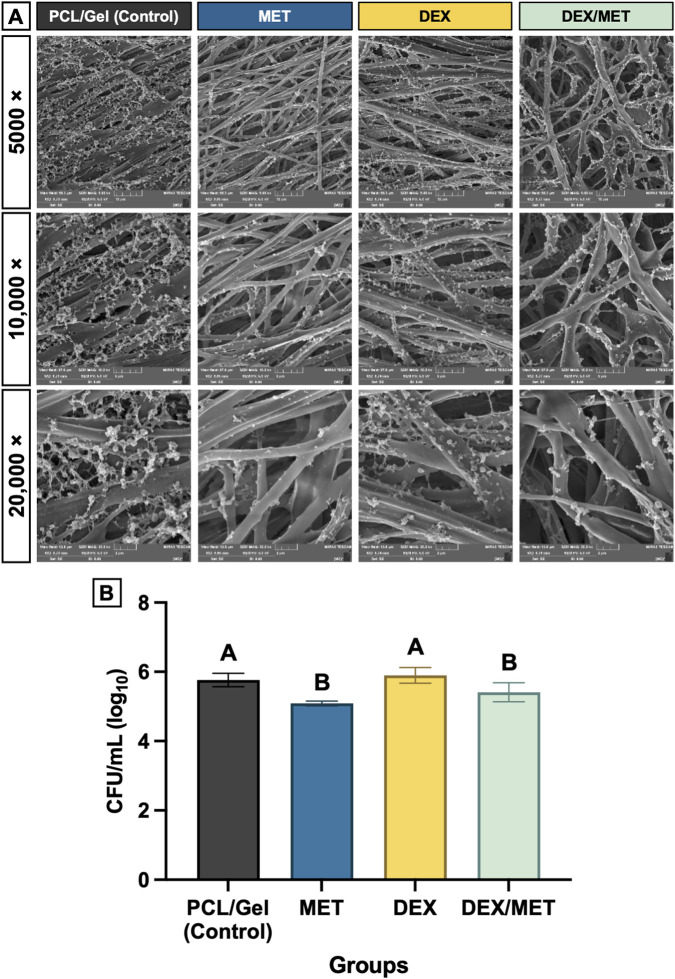
**(A)** SEM images of *Porphyromonas gingivalis* seeded on the scaffold surface after 4 days and **(B)** a graph representing the CFU/mL (log10) quantification. Mean and standard deviation. Different letters represent statistical differences between groups (ANOVA/Tukey; n = 6).

Incorporation of metronidazole (MET and DEX/MET groups) visibly impaired *P. gingivalis* (Pg) biofilm development on the scaffold surface. SEM imaging at 20,000× after 4 days of bacterial seeding showed less mature biofilm coverage and a more disrupted, disorganized biofilm structure on MET-containing scaffolds than on CT and DEX (Figure 4A). These qualitative findings were supported by CFU quantification. While the MET and DEX/MET groups demonstrated no significant differences with each other, both MET-containing groups exhibited significantly reduced bacterial recovery compared with scaffolds without MET. The MET group demonstrated a highly significant decrease in CFUs compared with CT and DEX (p < 0.0001). Similarly, the DEX/MET group also exhibited significantly lower CFU counts than CT and DEX (p ≤ 0.032) (Figure 4B).

### 3.3 aBMSC viability, spreading, and mineralized matrix formation on DEX/MET-loaded PCL/Gel core-shell scaffolds

**FIGURE 5:**
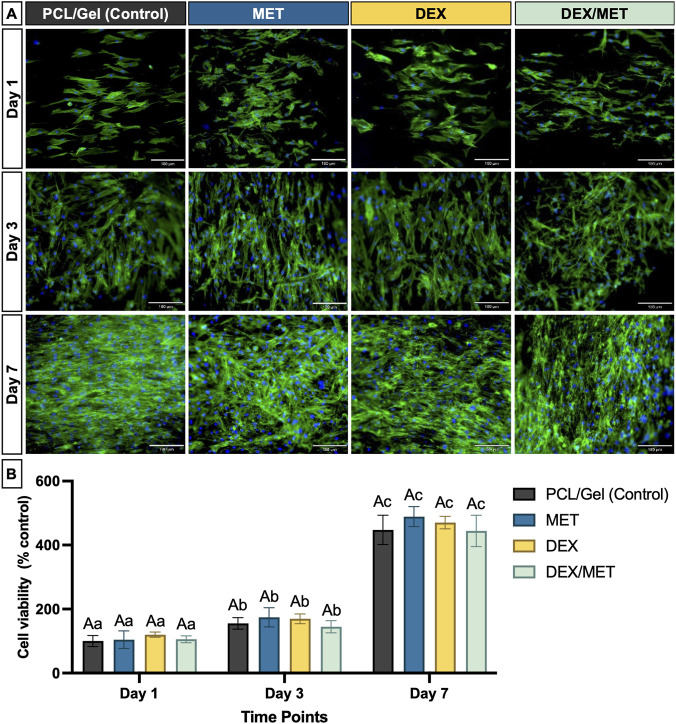
**(A)** Direct fluorescence images (10×) of aBMSCs seeded on the surface of the scaffolds. Actin filaments (Green) were stained with ActinGreen 488 reagent, and cell nuclei (blue) were labeled with DAPI. Scale bar = 180 μm; **(B)** Viability of aBMSCs seeded on the surface of the scaffolds. Mean and standard deviation. Capital letters indicate significant differences between groups; lowercase letters demonstrate significant differences between timepoints. (repeated measures ANOVA/Sidak; α = 5%, n = 10).

**FIGURE 6:**
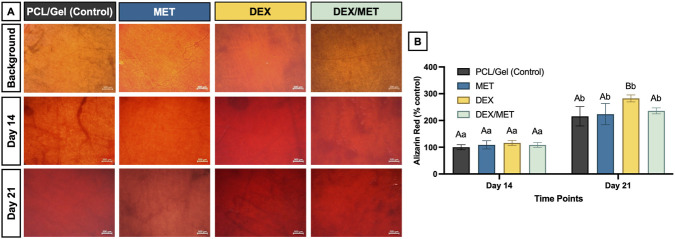
**(A)** Representative images of the alizarin red-stained scaffolds’ surface. **(B)** Mineralized matrix formation of aBMSCs seeded on the surface of the scaffolds. Mean and standard deviation. Capital letters indicate significant differences between groups; lowercase letters demonstrate significant differences between time points. (Two-way ANOVA/Sidak; α = 5%, n = 6).

Across all scaffold formulations, alamarBlue® results showed a steady increase in aBMSCs viability from Day 1 to Day 7, indicating that the scaffolds supported cell survival and metabolic activity over time (Figure 5B). Consistent with these findings, F-actin staining revealed progressive cell spreading on the scaffold surface, with more extensive cytoskeletal organization and evidence of increasing extracellular matrix coverage by Day 7 in all groups (Figure 5A). Mineralized matrix deposition increased over time in every group, with significantly higher mineralization at Day 21 compared with Day 14 (p < 0.0001) (Figures 6A,B). While all scaffolds supported osteogenic mineralization, the DEX group showed the strongest response: at Day 21, mineral deposition in the DEX group was significantly higher than in the other scaffold groups (p ≤ 0.0032) (Figures 6A,B).

### 3.4 Dexamethasone-loaded scaffolds attenuate LPS-induced inflammatory cytokine production

**FIGURE 7:**
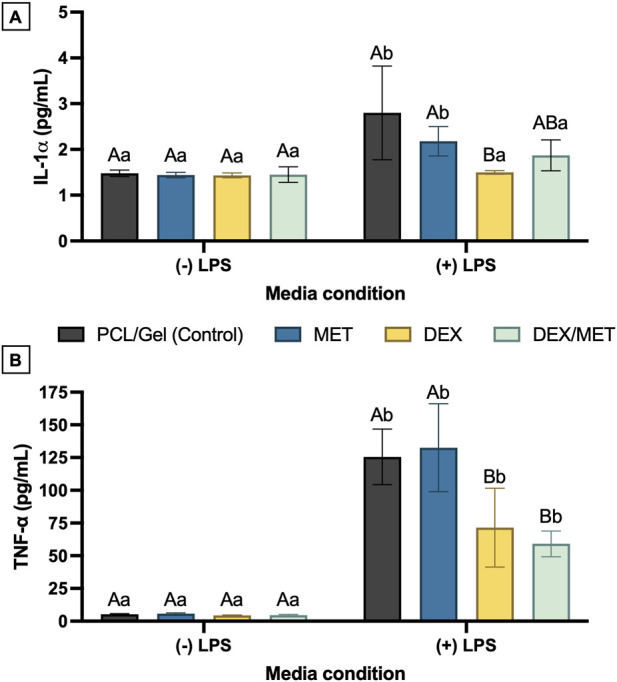
**(A)** IL1-α and **(B)** TNF-α synthesis by macrophages (RAW 264.7) stimulated or not by LPS and in contact with the scaffold extracts for 24 h. Mean and standard deviation. Capital letters indicate statistical differences between groups; lowercase letters indicate statistical differences between media conditions (Two-way ANOVA/Sidak; n = 6).

Cytokine profiling of RAW 264.7 macrophage cultures showed that scaffolds containing dexamethasone strongly modulated the inflammatory response following LPS stimulation. For IL-1α, levels returned to baseline only in the DEX and DEX/MET groups. In contrast, IL-1α remained significantly elevated in groups without DEX (CT and MET; p ≤ 0.0119) (Figure 7A). TNF-α increased significantly after LPS exposure in all groups, confirming a robust inflammatory challenge. Even so, dexamethasone-containing scaffolds markedly reduced TNF-α compared with CT and MET, with both DEX and DEX/MET showing significantly lower concentrations (p < 0.0001) (Figure 7B).

## 4 Discussion

Conventional barrier biomaterials used in guided tissue/bone regeneration are primarily designed to exclude epithelial and connective tissue ingrowth into defect sites, thereby preserving space for repopulation by regenerative cells (Bottino et al., 2012; Aytac et al., 2022). However, many of these materials are largely passive and lack therapeutic functionality, such as antibacterial activity or immunomodulation, which can limit regenerative outcomes in biologically hostile environments, such as periodontal defects (Bottino et al., 2012). In response, integrating bioactive agents into biomaterials through local-delivery strategies has emerged as a promising approach to enhance repair while reducing systemic exposure and side effects (Aytac et al., 2022; Passos et al., 2020). Core-shell architectures are particularly attractive because they enable spatial separation of drugs within distinct polymer compartments, creating opportunities for controlled and potentially sequential release that better match the temporal phases of wound healing (Perez and Kim, 2015). Given the high prevalence and clinical complexity of periodontitis, biomaterials that combine structural support with localized antimicrobial and immunoregulatory functions are particularly attractive for periodontal regenerative procedures, where infection control and inflammation resolution are critical determinants of treatment success (Slots, 2017).

In the present study, we developed a multifunctional coaxial electrospun scaffold intended to provide temporary structural support while locally delivering metronidazole (MET) and dexamethasone (DEX) to address two key barriers to periodontal regeneration: persistent infection and chronic inflammation. The design rationale leveraged the complementary properties of the selected polymers: a mechanically robust, slowly degrading PCL core to support prolonged structural stability and sustain DEX delivery, and a more hydrophilic gelatin (Gel) shell to promote early tissue-material interactions and facilitate MET availability during the initial, bacteria-dominated phase of healing. Overall, the platform maintained physicochemical integrity after drug incorporation and demonstrated biologically relevant antibacterial and anti-inflammatory effects while supporting mesenchymal stem cell viability and spreading.

Electrospinning was selected as the fabrication method because it enables production of ECM-mimetic fibrous scaffolds with high surface area and tunable microstructure, while also accommodating drug incorporation and scalable manufacturing (Perez and Kim, 2015; Fischer et al., 2025). Importantly, electrospun scaffold properties are sensitive to solution composition and processing parameters, and small changes can influence fiber diameter, pore architecture, and ultimately drug transport and cell behavior (Mitropoulou et al., 2024; Munchow et al., 2015; Mahmoud et al., 2023; Daghrery et al., 2025). Here, the core-shell configuration was confirmed by TEM and fluorescence labeling, which showed distinct core and shell compartments, supporting successful coaxial fiber formation. SEM further demonstrated continuous, uniform fibers across all groups, suggesting that drug loading did not compromise gross fiber formation. The observed variations in fiber diameter among the groups can be attributed to changes in solution properties induced by drug incorporation, including the presence of metronidazole and/or dexamethasone. It is well established that the addition of drugs to electrospinning solutions can alter key parameters such as viscosity, conductivity, and surface tension, thereby affecting jet stability and fiber morphology (Dragar et al., 2024). Incorporating metronidazole into polymeric systems have demonstrated that increasing drug concentration can decrease solution viscosity while increasing conductivity (Daghrery and Bottino, 2022; Bottino et al., 2017a; Ferreira et al., 2021). Although specific reports on dexamethasone are more limited, its incorporation as a small molecule is also expected to influence solution physicochemical properties in a similar manner. Therefore, small differences in drug loading and composition can explain the observed variations in fiber diameter, despite constant processing parameters.

FTIR spectra were consistent with the expected chemical composition and supported the presence of both PCL and gelatin across all scaffold formulations. However, no distinct peaks were exclusively attributable to DEX or MET, likely due to overlapping signals and their relative concentration. Moreover, the overall spectral profiles did not indicate chemical degradation or the formation of new covalent bonds. This suggests that drug incorporation occurred without altering the primary chemical structure, supporting a predominantly physical interaction within the scaffold system. This interpretation is further supported by the morphological and biological findings, as the preservation of the chemical composition is consistent with the maintained fiber architecture and the observed cellular responses. Together, these findings indicate that drug incorporation was achieved while preserving the chemical composition of the scaffolds and maintaining their biological functionality.

A key requirement for membranes or scaffolds used in periodontal regenerative procedures is functional stability throughout the healing period, given that bone formation and remodeling are prolonged processes (Mansour et al., 2023; Bottino et al., 2012; Aytac et al., 2022). In this study, all scaffolds exhibited slow mass loss in PBS, retaining ∼80% of initial mass over 2 months, consistent with a construct designed to remain present during early-to-intermediate healing. The observed stability likely reflects the inherent slow-degrading nature of PCL (Mndlovu et al., 2024) combined with stabilization of the gelatin shell via EDC crosslinking, which can reduce gelatin solubility and improve wet stability. Notably, drug incorporation appeared to modestly reduce degradation; however, without direct measurements of water uptake, porosity, or drug release, the mechanism should be interpreted cautiously. Drug loading can alter microstructure and diffusion pathways, which may either accelerate or retard degradation depending on the drug-polymer interactions and compartmental localization (Visan et al., 2021). Future studies that directly quantify release kinetics and scaffold microstructural changes during degradation will be important to connect mass-loss behavior to therapeutic availability over time.

From a handling and application standpoint, mechanical integrity is also essential, particularly for scaffolds intended to function as defect-site barriers or space-maintaining implants. Mechanical testing showed comparable Young’s modulus, tensile strength, and elongation across groups, indicating that incorporation of MET and/or DEX did not compromise bulk performance. The only significant difference detected was an increase in tensile strength in the DEX/MET group relative to the control. This effect is consistent with prior observations that certain small molecules can influence polymer chain packing and mechanical response, potentially by modifying crystallinity or intermolecular interactions in semi-crystalline systems (Arbeiter et al., 2021). While the magnitude of change was small, maintaining (or slightly improving) tensile behavior after drug loading is advantageous for clinical translation, where manipulation, trimming, and placement require predictable material performance.

Previous studies have investigated the mechanical properties of PCL/gelatin-based scaffolds fabricated by conventional electrospinning for periodontal tissue engineering applications (Yao et al., 2016; Aminmansour et al., 2024). Similar tensile strength and elongation at break values were reported for 1:1 PCL/gelatin ratios compared with the scaffolds designed (Yao et al., 2016). When PCL was combined with methacrylated gelatin (GelMA) at a 1:1 ratio, similar tensile strength values, lower elongation at break, and a higher Young’s modulus were observed, underscoring the role of gelatin methacrylation, crosslinking approach, and electrospinning configuration (coaxial versus conventional) in governing scaffold mechanical behavior (Aminmansour et al., 2024).

While these findings provide important insight into the mechanical performance of such systems, the degradation behavior and the ability to provide sustained and spatially controlled release of therapeutic agents are more clinically relevant parameters than absolute mechanical strength (Woo et al., 2021). Indeed, for adjunctive antimicrobial therapy in periodontal applications, maintaining scaffold integrity during the early healing phase while enabling gradual degradation and drug release are critical characteristics, which were demonstrated by the scaffolds developed (Woo et al., 2021).

This behavior can be further explained by the structural organization of the core-shell fibers. The mechanical behavior and degradation of the core-shell membranes are mainly governed by the PCL core, which degrades slowly. Since overall degradation during the evaluated period was limited (∼20% mass loss), the main load-bearing structure remained intact. This explains why mechanical properties were largely preserved across all groups, even though early degradation mainly involves the gelatin shell and surface features.

Because persistent bacterial contamination can sustain inflammation, disrupt matrix remodeling, and impair healing (Bowler et al., 2001; Guo and Dipietro, 2010), we first evaluated antibacterial performance utilizing *P. gingivalis* (Pg). Pg is a Gram-negative, anaerobic bacterium that plays a central role in periodontitis by shifting the oral microbiome toward a dysbiotic state (Hussain et al., 2015), leading to chronic inflammation and tissue breakdown. Therefore, evaluating the antibacterial efficacy of MET-incorporated scaffolds is a critical step in elucidating the membranes’ potential to prevent infection and support tissue regeneration. MET-containing scaffolds (MET and DEX/MET) reduced Pg biofilm formation, evidenced qualitatively by disrupted biofilm architecture on SEM and quantitatively by lower CFU recovery compared with groups lacking MET. These data support the premise that incorporating MET into the gelatin shell can provide early antibiofilm functionality and help establish a more permissive microenvironment for regeneration, consistent with prior work demonstrating MET activity against anaerobic periodontal pathogens (Reise et al., 2012; Silverberg et al., 2025). At the same time, the magnitude of CFU reduction suggests that antibacterial performance could still be strengthened. This may reflect factors such as biofilm maturity, diffusion limits in a dense matrix, or suboptimal local availability, depending on release kinetics. Optimization of MET loading, shell crosslinking density, and/or incorporation of complementary antimicrobial strategies may further improve antibiofilm efficacy, particularly under more challenging polymicrobial biofilm conditions.

In light of these findings, a limitation of the present study is that antibacterial performance was evaluated using a single-species model (*P. gingivalis*), which does not fully represent the polymicrobial nature of periodontal biofilms. *P. gingivalis* was selected as a representative keystone pathogen due to its key contribution to the development of periodontitis, where it compromises host defense mechanisms, promotes the overgrowth of commensal oral bacteria, and shifts the host response from a protective surveillance state to an exacerbated, destructive inflammatory reaction (Curtis et al., 2025). However, periodontal infections involve complex multispecies communities, including organisms such as *Fusobacterium nucleatum* and other anaerobic bacteria that contribute to biofilm structure and pathogenic synergy (Chen et al., 2022; Oogai et al., 2025). Therefore, while the current findings provide initial evidence of antibacterial efficacy, they may not capture the scaffold’s full performance in the complex environment of the oral cavity. Future studies will expand antimicrobial testing to include additional periodontal pathogens and more complex multispecies biofilm models to better evaluate the scaffold’s effectiveness in replicating *in vivo* oral conditions.

Cytocompatibility assays using aBMSCs demonstrated that all scaffold groups supported increasing cell viability over 7 days, along with progressive spreading and surface coverage as assessed by fluorescence imaging. These findings are consistent with the expected benefits of incorporating gelatin, which provides cell-interactive motifs and improves surface wettability relative to PCL alone (Echave et al., 2019; Wu et al., 2024). Importantly, no cytotoxic effects were observed with DEX incorporation at 5% (w/w in the PCL solution) under the conditions tested, despite prior reports that high concentrations of DEX can negatively affect periodontal cell types (Kim et al., 2013). Collectively, the viability and spreading results indicate that the PCL/Gel core-shell architecture, together with the chosen drug concentrations, created a permissive surface for early stem cell responses relevant to periodontal tissue repair.

Beyond cytocompatibility, controlling inflammation is central to periodontal regeneration because excessive cytokine signaling can impair osteogenesis and promote catabolism. In macrophage cultures, DEX-containing scaffolds (DEX and DEX/MET) markedly attenuated LPS-induced pro-inflammatory cytokine production. IL-1α returned to baseline only in DEX-containing groups, while remaining elevated in groups without DEX. Similarly, although TNF-α increased after LPS exposure across all groups, levels were significantly lower in DEX and DEX/MET than in CT and MET. These findings align with the known immunomodulatory role of glucocorticoids and support DEX as a local strategy to mitigate inflammatory signaling (Bordini et al., 2021; Noory et al., 2025). This is particularly relevant because IL-1α and TNF-α are key mediators in periodontal tissue destruction, contributing to leukocyte recruitment, collagen degradation, and osteoclast-driven bone loss (Nikolopoulos et al., 2008; Madureira et al., 2018; Papathanasiou et al., 2020). Notably, MET alone did not reduce these cytokines, consistent with the notion that antimicrobial activity does not necessarily translate into direct immunosuppression in acute inflammatory challenge models (Silverberg et al., 2025).

In osteogenic conditions, all groups exhibited increased mineral deposition from Day 14 to Day 21, indicating that the scaffold platform supported mineralized matrix formation over time. The DEX-only scaffold showed the strongest mineralization response at Day 21, consistent with DEX’s established role in promoting osteogenic differentiation and maturation (Yuasa et al., 2015; Daghrery et al., 2020; Noory et al., 2025). Interestingly, the combined DEX/MET scaffold did not show the same enhancement, suggesting that co-incorporation may attenuate the osteoinductive effect observed with DEX alone. Several mechanisms could contribute to this outcome. MET may exert dose- or context-dependent effects on osteogenic signaling, or it may indirectly alter the local availability of DEX by changing diffusion pathways, microstructure, or compartmental release behavior in the coaxial fibers. Alternatively, interactions between drug payloads and polymer phases could influence DEX partitioning within the semi-crystalline PCL core, where release often begins from amorphous regions (Zamani et al., 2017). While these hypotheses are plausible, the underlying mechanism remains unresolved and warrants targeted investigation, including direct measurement of release kinetics for each drug and mechanistic readouts of osteogenic pathways.

This study has limitations that should be considered when interpreting the findings. Most notably, encapsulation efficiency was not characterized, which may lead to an inaccurate estimation of the final drug-polymer ratio. In addition, drug release profiles were not quantitatively measured, which limits direct linkage between scaffold architecture and the observed biological outcomes. Future work using HPLC or LC-MS to quantify MET and DEX release over time would enable correlation of drug availability with antibiofilm effects, cytokine modulation, and osteogenic outcomes. Therefore, a comprehensive evaluation of drug-loading efficiency followed by detailed release kinetics would provide stronger evidence for optimal dosing and temporal delivery at the injury site to improve therapeutic efficacy while minimizing side effects. Furthermore, as this study provides an initial characterization of the core-shell fibers, certain material properties remain to be further examined. Future investigations employing swelling kinetic studies and degradation assessments across a range of pH levels would provide a more comprehensive profile of the scaffold’s structural stability and environmental responsiveness. In addition, antibacterial testing was performed against a single species *in vitro*, whereas periodontal disease is driven by polymicrobial dysbiosis; more complex multispecies biofilms and clinically relevant models would strengthen translational relevance. Moreover, while the current study quantitatively demonstrates the anti-inflammatory potential of the DEX-loaded scaffolds, further investigations using immunofluorescence (IF) staining, immunohistochemistry (IHC), and gene expression analysis are crucial. Such analyses will be vital for elucidating the spatial distribution of inflammatory markers and for fully understanding the material’s localized mechanistic role in modulating the scaffold-cell interface. Finally, *in vivo* validation in periodontal defect models will be essential to determine whether the combined antibacterial and anti-inflammatory effects translate into improved regeneration under physiological mechanical loading, immune complexity, and vascularization.

In summary, we demonstrate that coaxially electrospun PCL/Gel core-shell scaffolds can incorporate MET and DEX without compromising scaffold morphology, stability, or bulk mechanical performance, while providing distinct biological functionality: MET reduced *P. gingivalis* biofilm formation, and DEX attenuated LPS-induced macrophage cytokine production and enhanced mineralization when delivered alone. With further refinement, particularly in quantitative release characterization and in optimizing MET/DEX dosing and sequencing, this core-shell platform holds promise as a multifunctional local-delivery scaffold for periodontal regenerative therapy.

## 5 Conclusion

This study demonstrates that coaxially electrospun PCL/Gel core-shell scaffolds can incorporate metronidazole and dexamethasone while preserving core-shell architecture, structural stability, and mechanical performance. MET-containing scaffolds reduced *P. gingivalis* biofilm formation, and DEX-containing scaffolds attenuated LPS-induced macrophage cytokine production. All scaffold formulations supported aBMSCs viability and spreading, and mineralized matrix deposition increased over time, with the strongest osteogenic response observed in the DEX-only group. Collectively, these findings support PCL/Gel core-shell fibers as a multifunctional local-delivery scaffold with combined antibacterial and immunomodulatory activity and osteogenic cell compatibility, highlighting their promise for periodontal regenerative applications.

## Data Availability

The raw data supporting the conclusions of this article will be made available by the authors, without undue reservation.

## Supplementary material

SUPPLEMENTARY FIGURE S1 (A) Crosslinking protocol; (B) Remaining mass of experimental scaffolds in PBS over four months. Values are means and confidence intervals (95% CI, n = 6); (C) Viability (alamarBlue assay) of aBMSCs seeded on the surface of the crosslinked scaffolds. Values are mean and standard deviation. Capital letters indicate significant differences between groups; lowercase letters demonstrate significant differences between time points. (repeated measures ANOVA/Sidak post-hoc; α = 5%, n = 5); (D) Fourier Transform Infrared Spectroscopy (FTIR) analysis of core-shell scaffold degradation over 2-month period
