# Supplementary figures and images for: Coaxial electrospinning of poly(ɛ-caprolactone)/gelatin core-shell biodegradable implants for localized delivery of metronidazole and dexamethasone for periodontal applications
Source: Front Bioeng Biotechnol. 2026 May 22;14:1770271. doi: 10.3389/fbioe.2026.1770271 (PMC13237788; doi:10.3389/fbioe.2026.1770271)

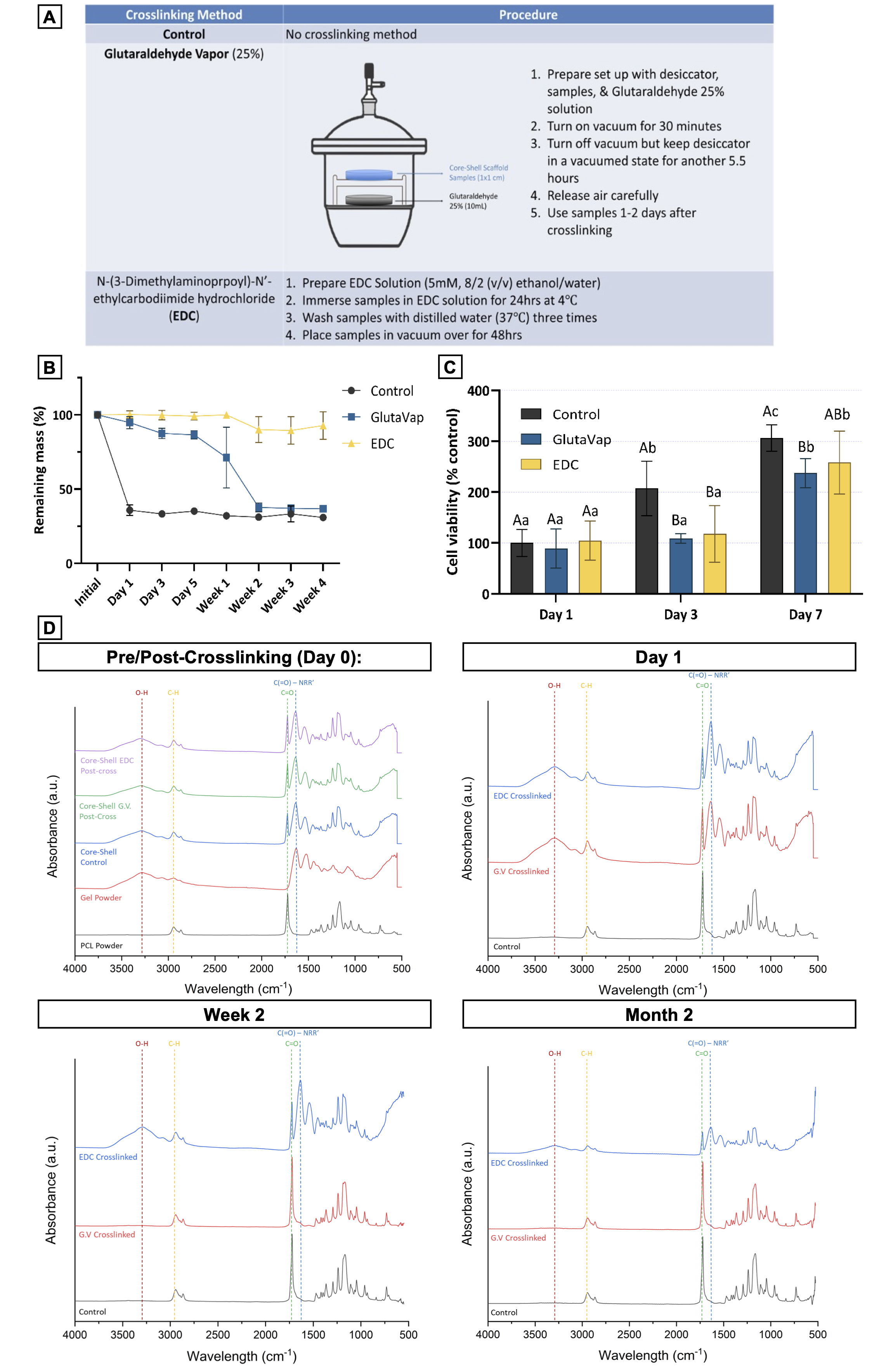

Supplement: Supplementary file 1 [file Image1.tiff]
